# Complete Genome Sequence of Megamonas funiformis Strain 1CBH44, Isolated from Human Feces

**DOI:** 10.1128/MRA.00785-21

**Published:** 2021-12-02

**Authors:** Yusuke Ogata, Mitsuo Sakamoto, Naveen Kumar, Moriya Ohkuma, Masahira Hattori, Wataru Suda

**Affiliations:** a Laboratory for Microbiome Sciences, RIKEN Center for Integrative Medical Sciences, Yokohama, Kanagawa, Japan; b Microbe Division/Japan Collection of Microorganisms, RIKEN BioResource Research Center, Tsukuba, Ibaraki, Japan; c PRIME, Japan Agency for Medical Research and Development, Tsukuba, Ibaraki, Japan; d Graduate School of Advanced Science and Engineering, Waseda University, Tokyo, Japan; University of Rochester School of Medicine and Dentistry

## Abstract

Here, we report the complete genome sequence of Megamonas funiformis strain 1CBH44, which was isolated from the feces of a healthy Japanese person. The genome consists of a circular chromosome (2,310,709 bp, with a GC content of 31.5%) and possesses 2,170 putative protein-coding genes, 18 rRNA genes, and 54 tRNA genes.

## ANNOUNCEMENT

Megamonas funiformis is an anaerobic, nonmotile, non-spore-forming, Gram-negative-staining member of the human gut microbiota (1). The complete genome of the type strain (M. funiformis JCM 14723) has been reported (2), and here we report the genome of an additional strain, M. funiformis strain 1CBH44, which was isolated from the feces of a healthy Japanese person.

The manufacturer’s instructions and default parameters were used for all products and software, respectively, unless otherwise specified. Collection of the fecal sample and the experiments were conducted in accordance with RIKEN ethics committee approval (approval no. Tsukuba 27-1). A fecal sample (0.5 g) was suspended in 4.5 ml of prereduced phosphate-buffered saline (PBS). The diluted fecal sample was plated on Columbia blood agar supplemented with 5% (vol/vol) horse blood for 2 to 4 days of incubation at 37°C in an environment containing a gas mixture of H_2_/CO_2_/N_2_ (1:1:8, by volume). M. funiformis strain 1CBH44 was identified and isolated by 16S rRNA gene sequencing as described previously (3). The purified 1CBH44 strain was then grown in 500 ml of Gifu anaerobic medium (GAM broth; Nissui) for 2 days at 37°C to prepare the genomic DNA.

The genomic DNA was extracted with enzymatic lysis as reported previously (4). The DNA sequencing was performed using two platforms, i.e., the Illumina MiSeq platform (2 × 300-bp paired-end reads) and the Pacific Biosciences (PacBio) Sequel platform; the libraries were prepared using the TruSeq DNA PCR-free kit (550-bp target length) and the SMRTbell template preparation kit v2.0 (10- to 15-kb target length) without DNA shearing, respectively. The MiSeq reads were trimmed and filtered with a quality value (QV) of >20 using FASTX-toolkit (v0.0.13) (hannonlab.cshl.edu/fastx_toolkit), and error correction of the Sequel reads was performed using Canu (v2.1.1) (5) with additional options as described previously (6). The *de novo* hybrid assembly of quality-checked reads and the quality check of the generated genome for overlapping and circularization were performed using Unicycler (v0.4.8) (7).

We obtained a total of 1,493,235,959 bases from 2,495,236 filter-passed MiSeq paired-end reads, with an average length of 299.2 bp, and a total of 1,330,191,598 bases from 137,891 corrected PacBio reads, with an *N*_50_ value of 11,968 bp. The hybrid assembly generated a circular contig with an average read depth of 1,222×.

The M. funiformis strain 1CBH44 chromosome is 2,310,709 bp in length, with a GC content of 31.5%, and contains 2,170 protein-coding genes, 54 tRNA genes, six 5S rRNA genes, six 16S rRNA genes, and six 23S rRNA genes, as determined by DFAST (v1.2.4) (dfast.nig.ac.jp) (8). The 16S rRNA sequence had the greatest similarity to that of M. funiformis JCM 14723^T^, with a 99.5% identity match in the NCBI nucleotide database (21 June 2021), and the average nucleotide identity (ANI) between the genomes of 1CBH44 and JCM 14723^T^ was estimated to be 97.9% using fastANI (v1.32) (9). Comparison of the two genomes revealed that the 1CBH44 genome was 8.4% smaller than that of JCM 14723^T^ (2,522,577 bp), including five deletions of >50 kb, three of which contained phage-related proteins unique to the 1CBH44 genome; there were five insertions of >10 kb, which were located within other regions of the five deletions of >50 kb.

### Data availability.

The complete genome sequence of Megamonas funiformis strain 1CBH44 was deposited in DDBJ/ENA/GenBank under the accession no. AP024966, which is linked to the BioProject accession no. PRJDB12036, the BioSample accession no. SAMD00393850, and the DDBJ Sequence Read Archive (DRA) accession no. DRX299965 and DRX299966.
